# Negative Allosteric Modulation of Agonist-Induced M_2_ Muscarinic Receptor/β-Arrestin Interaction by Serum Autoantibodies from Patients with Chronic Chagas Disease

**DOI:** 10.3390/cells14231857

**Published:** 2025-11-25

**Authors:** Laura C. Carrera Páez, Sabrina P. Beltrame, Sergio R. Auger, Ahmad H. Sabra, Claudio R. Bilder, Isabel M. Irurzun, Claudia I. Waldner, Juan C. Goin

**Affiliations:** 1 Laboratorio de Farmacología Molecular, Centro de Estudios Farmacológicos y Botánicos (CEFYBO-CONICET-UBA) y II Cátedra de Farmacología, Facultad de Medicina, Universidad de Buenos Aires, Buenos Aires C1121ABG, Argentina; lcarrerapaez@chu-besancon.fr (L.C.C.P.); beltri12@yahoo.com.ar (S.P.B.); 2 Hospital Donación Francisco Santojanni, Buenos Aires C1408INH, Argentina; sergioauger@aol.com (S.R.A.); amsabra@intramed.net (A.H.S.); 3 Laboratorio de Neurogastroenterología, Fundación Favaloro-Hospital Universitario, Buenos Aires C1093AAS, Argentina; cbilder@ffavaloro.org; 4 Centro de Simulación Computacional para Aplicaciones Tecnológicas (CSC-CONICET), Buenos Aires C1425FQD, Argentina; i_irurzun@hotmail.com; 5 Laboratorio de Inmunidad Celular y Molecular, Centro de Estudios Farmacológicos y Botánicos (CEFYBO-CONICET-UBA), Facultad de Medicina, Universidad de Buenos Aires, Buenos Aires C1121ABG, Argentina; claudiawaldner@conicet.gov.ar

**Keywords:** neglected tropical disease, *Trypanosoma cruzi*, autoimmunity, neurotransmitter receptor, signal transduction, biased ligand, dysautonomia, heart disease, pathophysiology, BRET

## Abstract

**Highlights:**

**What are the main findings?**

- Circulating autoantibodies to M_2_ muscarinic receptors from patients with chronic Chagas disease can inhibit the interaction of agonist-induced arrestin-2, but not arrestin-3.
- These antibodies can act as negative allosteric modulators of agonist efficacy.

**What are the implications of the main finding?**

- Allosteric inhibition of agonist-induced M_2_ receptor activation by autoantibodies could play a pathogenic role in cardiac parasympathetic dysfunction secondary to Chagas disease. These results support the potential therapeutic use of allosteric ligands to treat pathogenic effects of functional autoantibodies to M_2_ muscarinic receptors in patients with chronic Chagas disease and other pathological conditions.

**Abstract:**

Inhibition of agonist-induced M_2_ muscarinic receptor (M_2_R) activation by functional anti-M_2_R autoantibodies has been associated with cardiac parasympathetic dysfunction in patients with chronic Chagas disease (CD). This study explored the allosteric nature of that inhibitory effect by assessing the ability of serum IgG from patients with CD and dysautonomia (DCD IgG) to modulate the interaction between M_2_R and β-arrestins in HEK 293T cells using bioluminescence resonance energy transfer. DCD IgG alone did not stimulate arrestin-2 or arrestin-3 recruitment. When cells were preincubated with DCD IgG and then treated with carbachol, arrestin-2 translocation decreased in a concentration-dependent manner, while arrestin-3 recruitment remained unaffected. Inhibition curve analysis showed a submaximal inhibitory effect (68.1 ± 2.4%) and a Hill slope less than −1 (−4.03 ± 0.39). Carbachol concentration–response assays after preincubation with DCD IgG revealed a noncompetitive inhibition of arrestin-2 recruitment, with no change in arrestin-3 translocation. Unlikely, simultaneous exposure to DCD IgG and carbachol potentiated agonist-induced Arr-2 recruitment. We conclude that anti-M_2_R autoantibodies selectively inhibit agonist-induced arrestin-2 recruitment, acting as negative allosteric modulators of agonist efficacy. The direction of autoantibody-induced allosteric modulation depends on the timing of IgG application relative to the agonist and the duration of receptor exposure to autoantibodies.

## 1. Introduction

Chagas disease (CD) is a neglected tropical parasitic disease caused by the protozoan *Trypanosoma cruzi* (*T. cruzi*). The infection primarily occurs in endemic areas of Latin America but spreads to other non-endemic countries in the Americas and worldwide due to the migration of infected individuals [1]. The course of CD comprises a brief acute phase characterized by high parasitemia and usually mild, nonspecific symptoms, and a chronic phase with low or undetectable parasitemia and high levels of circulating anti-*T. cruzi* antibodies. While most infected individuals remain asymptomatic for life, around 30% of them experience cardiac disorders such as conduction abnormalities, arrhythmias, dilated cardiomyopathy, or heart failure, among other complications. Besides, less than 10% of all infected patients suffer from digestive disorders, and under 5% develop neurological forms of the disease [2].

Functional autoantibodies (AAb) against β_1_ and β_2_ adrenergic receptors (β_1_AR/β_2_AR) [3,4,5] and M_2_ muscarinic acetylcholine receptors (M_2_R) have been identified in patients with chronic CD. The interaction of these antibodies with their main epitopes at the second extracellular loop (II-ECL) of their corresponding receptors promotes receptor activation and triggers receptor-specific G protein-mediated signal transduction. The main epitopes from β_1_AR and M_2_R recognized by functional AAb share structural similarities with *T. cruzi* ribosomal proteins. Hence, these AAb are believed to be the result of an autoimmune response against the parasite through a molecular mimicry mechanism [6,7,8].

Anti-βAR AAb are highly prevalent in patients with chronic CD and ventricular tachycardia [9], while anti-M_2_R AAb correlate with sinus node dysfunction [9], esophageal achalasia [10], megacolon [11], and increased ventricular repolarization heterogeneity [12]. In addition, a strong association between M_2_ muscarinic AAb and cardiac dysautonomia has been reported in asymptomatic or symptomatic individuals with CD [13]. Moreover, the levels of serum anti-M_2_R AAb correlate with the degree of parasympathetic impairment as determined by reduced high-frequency power in heart rate variability [14]. These results suggest that these AAb could be used as diagnostic biomarkers for parasympathetic dysautonomia in CD.

Due to growing evidence suggesting that anti-M_2_R AAb may be involved in the pathophysiology of cardiac parasympathetic inhibition, we hypothesized that these AAb could impair acetylcholine (ACh)-mediated signaling transduction by attenuating M_2_R activation. Accordingly, we found that exposure of M_2_R expressed in HEK 293T cells to anti-M_2_R AAb, followed by the addition of carbachol, promotes the inhibition of agonist-induced Gi protein activation and arrestin-2 (Arr-2) recruitment to the M_2_R [15].

As regards the mechanism underlying the inhibitory effect of anti-M_2_R AAb on agonist-induced M_2_R activation, most previous reports suggest that these AAb cannot promote M_2_R desensitization or internalization [15,16,17]. Thus, the inhibitory effect of anti-M_2_R is unlikely to be the result of short-term receptor regulation. Alternatively, several studies support the notion that an allosteric mechanism could mediate this effect: (a) The II-ECL of the M_2_R, which interacts with anti-M_2_R AAb, contributes to the common allosteric binding site for muscarinic allosteric ligands. This means that muscarinic AAb interact with the main allosteric site of the M_2_R [18]; (b) Anti-M_2_R AAb exert a non-competitive inhibition of muscarinic antagonist binding to the M_2_R, which is consistent with an allosteric antibody/receptor interaction [5,19]; (c) Anti-M_2_R AAb enhance M_2_ muscarinic receptor/receptor interaction through receptor crosslinking. The fact that the negative allosteric modulator (NAM) gallamine attenuates this effect suggests that gallamine competes with muscarinic AAb for binding to the receptor’s allosteric binding site [20].

The data discussed above are consistent with the potential allosteric nature of the inhibitory effect of anti-M_2_R AAb on agonist-induced M_2_R activation. However, this hypothesis appears to contradict previous results reported by Hernández et al., who demonstrated that serum AAb from patients with CD can enhance ACh-induced negative chronotropism in isolated rabbit hearts, suggesting that anti-M_2_R AAb can function as positive allosteric modulators (PAM) [17].

The general aim of our research was to elucidate the pharmacological basis for the role of anti-M_2_R AAb in cardiac parasympathetic inhibition secondary to CD. Accordingly, we hypothesized that the impairment of agonist-induced M_2_R activation by such antibodies is mediated by negative allosteric modulation. In this study, the allosteric nature of the inhibitory effect of muscarinic AAb from patients with CD on agonist-induced β-arrestin recruitment to the M_2_R was examined in heterologous HEK 293T cells by bioluminescence resonance energy transfer (BRET). In addition, both the mode of allosteric inhibition and the ability of these AAb to act as PAM were explored. Finally, the pharmacological implications of our findings in the context of the pathophysiology of cardiac CD were addressed.

## 2. Materials and Methods

### 2.1. Patients

Patients with chronic *T. cruzi* infection (CD) and healthy noninfected individuals were recruited at Donación Francisco Santojanni Hospital (Buenos Aires, Argentina) upon referral from the hospital outpatient clinics. All volunteers resided in the Buenos Aires Metropolitan Area at the time of the study. However, those with *T. cruzi* infection had a history of previous residence in an endemic area for CD, at least 15 years before migrating to non-endemic areas. Diagnosis of CD was based on the detection of anti-*T. cruzi* antibodies in at least two standard tests using different antigens [indirect hemagglutination (IHA) and ELISA]. In the event of a discrepancy between the two results, a third test was performed [chemiluminescence microparticle immune assay (CMIA)]. This diagnostic strategy is based on the guidelines issued by the Pan American Health Organization in 2019 [21]. All volunteers participating in the study underwent comprehensive clinical and cardiovascular assessments, including a clinical history, physical examination, a standard 12-lead electrocardiogram (ECG), a 24 h Holter ECG, and 2D echocardiography.

Volunteers with chronic systemic diseases, any other infectious diseases, or those undergoing treatment with benznidazole were excluded from participation in this study. Patients who had received an organ transplant or blood transfusions were also excluded. *T. cruzi*-infected patients exhibited ECG disturbances like conduction abnormalities or arrhythmias typical of CD [16]. However, echocardiographic features, such as left ventricular dimension, global or regional wall motion, and left ventricular ejection fraction, ranged within normal values. The ability of the autonomic nervous system to regulate blood pressure and heart rate was assessed in all patients using a set of four classical tests (Valsalva maneuver and tilting, hyperventilation, and coughing tests) [22]. The diagnosis of dysautonomia in each patient was established based on at least two abnormal tests, according to standard procedures [23]. For our research, all volunteers were divided into two groups: Group 1: Patients with chronic CD and dysautonomia (DCD patients; *n* = 15), and Group 2: Control noninfected individuals (Control; *n* = 15). All DCD patients exhibited lower Valsalva ratios than age-matched normal values (cut-off value = 1.5), which has been associated with impaired parasympathetic modulation [23].

### 2.2. Reagents and Antibodies

Tetracycline hydrochloride, ampicillin sodium salt, kanamycin sulfate, Tween-20, p-nitrophenyl phosphate, tris (hydroxymethyl) aminomethane, gallamine triethiodide, atropine sulfate, carbamoylcholine chloride (carbachol), bovine serum albumin (BSA), and Folin–Ciocalteu’s phenol reagent were purchased from Sigma-Aldrich (St. Louis, MO, USA). Biotechnological fetal bovine serum (FBS) was procured from Internegocios S.A. (Mercedes, Argentina). Dulbecco’s modified Eagle medium with high glucose (DMEM) was purchased from Thermo Fisher Scientific (Waltham, MA, USA). Penicillin G sodium salt and streptomycin sulfate were provided by Richet Laboratories (Buenos Aires, Argentina). Diethylaminoethyl cellulose (DE-52) was purchased from Whatman (Maidstone, UK). Trypsin from bovine pancreas was purchased from Worthington (Lakewood, NJ, USA). Coelenterazine-h was obtained from Biotium (Fremont, CA, USA). Alkaline phosphatase-conjugated goat anti-human and goat anti-mouse IgG were obtained from Jackson Immuno Research (West Grove, PA, USA). The monoclonal antibody directed to the II-ECL of the human M_2_R (B8E5) was purchased from Novus Biologicals (Centennial, CO, USA).

### 2.3. ELISA

Sera from *T. cruzi*-infected and noninfected volunteers were tested for the presence and levels of anti-M_2_R Ab by indirect ELISA [24]. In this assay, a synthetic peptide within the II-ECL of the human M_2_R (pM_2_) was used as an immobilized antigen. The amino acid sequence of pM_2_ (VRTVEDGECYIQFFSNAAVTFGTAI) corresponds to residues 169–193 of the human M_2_R (CHRM2, Gene ID: 1129) [25]. Maxisorp microplates (Nunc, Waltham, MA, USA) were first coated with pM_2_ (2.5 µg/well) in 0.1 M Na_2_CO_3_ buffer, pH 11, for 18 h at 4 °C and then blocked with 2% BSA in phosphate-buffered saline, pH 7.4 (PBS) for 2 h at 37 °C. Next, 100 µL of human serum diluted 1:50 in dilution buffer (1% BSA in PBS) was added to the coated wells and allowed to react with the antigen for 2 h at 37 °C. Subsequently, 100 µL of alkaline phosphatase-conjugated goat anti-human IgG diluted 1:6000 in dilution buffer was added to each well, and the plate was incubated for 1 h at 37 °C. Three washes with PBS supplemented with 1% Tween-20 were performed between incubation steps. Finally, OD_405nm_ was measured after incubation with 1 mg/mL p-nitrophenyl phosphate for 30 min at room temperature. Serum immune reactivity was expressed as ELISA reactivity index (ERI), where ERI = Mean OD_405nm_ of each serum sample/(Mean OD_405nm_ + 2 SD of control sera) (*n* = 15). Sera with ERI values higher than 1 (cut-off value) were taken as positive for anti-M_2_R AAb.

The assay included negative controls such as the blank (only substrate and stop solution), nonspecific controls with the absence of primary antibody (human serum), and sera from three patients with Chagas disease and negative immune reactivity for anti-M_2_R AAb as determined in a previous study [15].

As positive controls, we included six sera from patients with Chagas disease, and high (*n* = 2), medium (*n* = 2), and low titers (*n* = 2) of anti-M_2_R AAb, as determined in a previous study [15]. In addition, we included a commercially available monoclonal antibody against the II-ECL of the human M_2_R (pM_2_) with a high titer of anti-M_2_R AAb as detected using an alkaline phosphatase-conjugated goat anti-mouse IgG antibody. All determinations were performed in duplicate.

### 2.4. Purification of the IgG Fraction from Human Serum

The serum IgG fractions from eight non-infected individuals seronegative for anti-pM_2_ AAb (Control IgG), and eight *T. cruzi*-infected patients with dysautonomia seropositive for anti-pM_2_ AAb (DCD IgG) (Mean ERI ± SEM = 2.20 ± 0.33) were purified by DEAE-cellulose chromatography, as described previously [24]. Sera were dialyzed overnight against the elution buffer (10 mM Na/K phosphate, pH 8) and then passed through DEAE-cellulose columns that had been previously equilibrated with the elution buffer. The eluted IgG peaks were concentrated by ultrafiltration (Amicon Ultrafiltration Cell, Model 8010, Millipore Corporation, Bedford, MA, USA) to approximately 10–15 mg/mL. The purity of concentrated IgG fractions was assessed by SDS-PAGE (8% T), while protein concentration in those samples was measured using the Lowry method [26].

### 2.5. Plasmids, Cell Culture, and Transfection

Several laboratories kindly provided plasmid constructs used throughout this study, as follows: M_2_R-RLuc (Dr. N.M. Nathanson, University of Washington, Seattle, WA, USA); Arr-2-YFP and Arr-3-YFP (Dr. M. Bouvier, University of Montreal, Montreal, QC, Canada); pCEFL-GRK2 (Dr. C. Shayo, IByME-CONICET, Buenos Aires, Argentina). All recombinant plasmids were amplified in XL1blue competent cells and purified using the Wizard^®^ Plus Maxiprep DNA Purification System (Promega, Madison, WI, USA). Human embryonic kidney cells (HEK 293T) (ATCC# CRL-3216) were provided by the American Type Culture Collection (Manassas, VA, USA). These cells were used throughout all BRET assays. These cells were cultured in Dulbecco’s modified Eagle’s medium (DMEM) (high glucose, 4.5 g/L) supplemented with 10% heat-inactivated FBS, 2 mM glutamine, 100 IU/mL penicillin G sodium salt, and 100 μg/mL streptomycin sulfate, and maintained at 37 °C in a humidified atmosphere with 5% CO_2_. Transient transfections were performed on 40–50% confluent cells using a standard calcium phosphate precipitation protocol [27].

### 2.6. BRET Assays

#### 2.6.1. Preparation of Cells Expressing BRET Constructs

A cell-based BRET^1^ system was used throughout this study. HEK 293T (HEKT) cells growing in 12-well plates were transiently transfected with the M_2_ muscarinic receptor fused to *Renilla* luciferase (M_2_R-RLuc), G protein-coupled receptor kinase 2 (GRK2), and arrestin-2 or arrestin-3 fused to the enhanced yellow fluorescence protein (Arr-2-YFP or Arr-3-YFP), yielding two alternative BRET cell systems (HEKT/Arr-2 and HEKT/Arr-3, respectively) [15,28]. After 48 h of incubation at 37 °C, cells from each well were washed twice with warm PBS and resuspended in 800 µL of a modified Krebs-Ringer-HEPES buffer (KRH) supplemented with bovine serum albumin (BSA) (KRHA: 130 mM NaCl, 5 mM KCl, 1.2 mM MgSO_4_, 1.2 mM CaCl_2_, 1.2 mM Na_2_HPO_4_, 10 mL glucose, 20 mM HEPES, 0·1% BSA, pH 7·4) [20].

#### 2.6.2. Assessment of Total Fluorescence, Total Luminescence, and Basal BRET Ratio

The expression of M_2_R-RLuc and Arr-2-YFP (Arr-3-YFP) in each cell suspension was monitored by measuring total luminescence and total fluorescence, respectively. The basal BRET signal between RLuc and YFP within each BRET pair was also measured. All determinations were performed using a FLUOstar Omega multi-mode microplate reader (BMG Labtech, Ortenberg, Germany).

(a) Fluorescence determinations: Ninety microliters of each cell sample was transferred to a 96-well black-walled, clear-bottomed microplate. Ten microliters of KRHA buffer was added to each well of the assay plate to complete a total volume of 100 µL. Total fluorescence was measured at 25 °C using an excitation filter of 485 nm and an emission filter of 535 nm.

(b) Luminescence determinations: Ninety microliters of each cell sample was transferred to a 96-well white opaque microplate. Ten microliters of a 10X coelenterazine h solution was added to each well to achieve a final substrate concentration of 5 µM. The plate was incubated at 25 °C in the dark. After 15 min of incubation, basal BRET determinations were taken by sequentially recording light intensity at 445–505 nm and 505–565 nm. Subsequently, total luminescence was measured 20 min after the addition of coelenterazine-h.

(c) Expression of results: Total fluorescence (Ft) as measured in relative fluorescence units (RFU), and total luminescence values (Lt), as measured in total luminescence units (RLU), were corrected by subtracting the background fluorescence (Fo) or background luminescence (Lo) determined in wells containing untransfected cells, respectively. As a result, corrected fluorescence (Fc) was calculated as Fc = Ft − Fo, while corrected luminescence (Lc) was calculated as Lc = Lt − Lo. The BRET ratio was calculated as BRET = Lc_505–565nm_/Lc_445–505nm_.

#### 2.6.3. BRET Protocols

Using F_c_ and L_c_ values from each transfected cell suspension, F_c_/L_c_ ratios were determined, and suspensions with similar F_c_/L_c_ ratios were pooled. Seventy microliters of pooled HEKT/Arr-2 or HEKT/Arr-3 cells were distributed in luminescence microplates.

Determination of Arr-2 or Arr-3 recruitment on the corresponding BRET cell system was performed at 25 °C using three different BRET procedures (a–c) [28]:

(a) To evaluate the effects of carbachol or IgG fractions on β-arrestin recruitment, cells were treated with carbachol, DCD IgG, or Control IgG for 20, 30, or 60 min.

(b) To assess the modulatory effect of muscarinic orthosteric or allosteric ligands, or IgG fractions, on agonist-induced Arr-2 or Arr-3 recruitment, cells were preincubated with atropine, gallamine, Control IgG, or DCD IgG fraction for 0, 15, 30, and/or 60 min. Then, carbachol was added, and cells were incubated for an additional 20 min.

(c) To assess the ability of a muscarinic peptide (pM_2_) to neutralize the inhibitory effect of DCD IgG on agonist-induced Arr-2 or Arr-3 translocation, DCD IgG fractions were first incubated with the muscarinic peptide for 30 min at 37 °C. Then, cells were preincubated with DCD IgG/pM_2_ cocktails for 30 min and further treated with carbachol for an additional 20 min.

Readings and Expression of Results (a–c): Fifteen minutes before the end of the total incubation time, 10 µL of 10X coelenterazine h solution was added to achieve a final substrate concentration of 5 µM at a final volume of 100 µL/well. Net BRET values were obtained following treatment with carbachol or IgG fractions, with or without pretreatment, and expressed as ∆BRET = (BRET ratio after treatment with carbachol or IgG fractions) − (BRET ratio after treatment with KRHA buffer alone). All ∆BRET values were multiplied by 1000 and expressed as millibrets (mB).

### 2.7. Statistical Analysis

Stimulation and inhibition concentration response curves were analyzed by nonlinear regression using a three-parameter logistic model with variable slope. Comparison among pharmacodynamic parameters was analyzed using the extra sum-of-squares F test or one-way ANOVA, followed by Bonferroni’s post hoc test. Corresponding equations, as well as suggestions for curve fitting and parameter definition, are shown in Supplementary Materials. Differences in the means of a continuous dependent variable across different groups defined by two categorical independent variables were analyzed using a two-way ANOVA, followed by Bonferroni’s post hoc test.

Throughout the present study, a *p*-value of 0.05 or less was considered statistically significant. The symbols *, &, or # are used as abbreviations in figures and tables to denote different degrees of statistical significance (*p*-value thresholds), with a greater number of symbols indicating a lower, more significant *p*-value. An asterisk (*) symbol corresponds to specific *p*-value thresholds as follows: *: *p* ≤ 0.05, **: *p* < 0.01, ***: *p* < 0.001, ****: *p* < 0.0001. For the ampersand (&) and hash (#) symbols, the same pattern of increasing significance with multiplicity is used. All statistical analyses were performed using the software GraphPad Prism v.8. (GraphPad, La Jolla, CA, USA).

## 3. Results

### 3.1. Agonist-Induced β-Arrestin Translocation in HEK 293T Cells. Orthosteric and Allosteric Modulation

The main goal of this study was to assess the allosteric nature of the inhibition of agonist-induced M_2_R/β-arrestin interaction promoted by anti-M_2_R AAb from DCD patients. Because the effect of these AAb on Arr-3 recruitment to the activated M_2_R had not been previously addressed, we first compared the abilities of Arr-2 and Arr-3 to interact with this receptor upon carbachol stimulation. In addition, the modulatory activity of these effects by orthosteric and allosteric ligands was examined.

**Figure 1:**
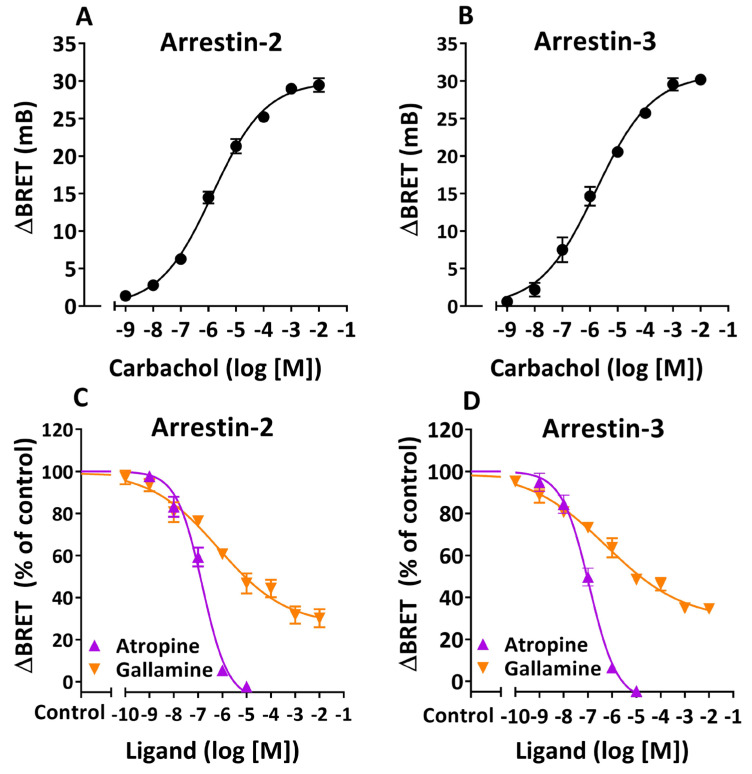
Characterization of agonist-induced β-arrestin recruitment in human cells expressing M_2_ muscarinic receptors. Inhibition by orthosteric and allosteric ligands. HEK 293T cells expressing M_2_R-RLuc, GRK2, and Arr-2-YFP (HEKT/Arr-2) or Arr-3-YFP (HEKT/Arr-3) were treated with carbachol at various concentrations for 20 min (**A**,**B**). Alternatively, either cell preparation (HEKT/Arr-2 or HEKT/Arr-3) was preincubated in the presence or absence of atropine or gallamine at multiple concentrations for 15 min, and then treated with carbachol (10 µM) for an additional 20 min (**C**,**D**). At the end of either procedure ((**A**)/(**B**) or (**C**)/(**D**)), BRET was determined in the presence of 5 µM coelenterazine h. Net changes in BRET were calculated as ΔBRET values and expressed in mB, where ΔBRET is the BRET ratio in the presence of agonist minus the BRET ratio in the presence of KRH buffer alone. (**A**,**B**) Data are expressed as ΔBRET (mB). (**C**,**D**) Data are expressed as a percentage of control ΔBRET values, which correspond to cells preincubated with KRH buffer alone and further treated with carbachol. Control values: 21.5 ± 0.8 mB (atropine) and 20.6 ± 1.2 mB (gallamine) (**C**); 20.5 ± 0.8 mB (atropine) and 20.2 ± 1.8 mB (gallamine) (**D**). Data shown are the mean ± SEM of four independent experiments performed at least in triplicate. All curves were best fit to a sigmoidal function using nonlinear regression analysis. Analysis of pharmacodynamic parameters derived from the data plotted in (**C**,**D**) is presented in Table 1.

Treatment of HEKT/Arr-2 or HEKT/Arr-3 cells with carbachol at varying concentrations resulted in concentration-dependent increases in the BRET signal between RLuc and YFP, indicating that either Arr-2 or Arr-3 is recruited to the M_2_R upon agonist exposure (Figure 1A,B). Comparison of pharmacodynamic parameters E_max_ and pEC_50_ between HEKT/Arr-2 and HEKT/Arr-3 cells using the extra sum of squares F test showed no significant differences [E_max_ (mB): 29.9 ± 0.6 (Figure 1A), 30.9 ± 0.9 (Figure 1B); pEC_50_: 5.85 ± 0.08 (Figure 1A), 5.78 ± 0.11 (Figure 1B)]. The fact that the efficacy and potency values of carbachol acting on both cell systems are not significantly different suggests that the translocation of Arr-2 and Arr-3 is governed by a shared mechanism (Figure 1A,B).

Subsequently, either cell system was preincubated with atropine (a muscarinic antagonist) or gallamine (a muscarinic NAM) at varying concentrations for 15 min, and further treated with carbachol at a 10 µM final concentration for an additional 20 min. Both ligands inhibited carbachol-induced translocation of either β-arrestin in a concentration-dependent manner (Figure 1C,D). In particular, atropine promoted complete inhibition of agonist-induced M_2_R/Arr-2 or M_2_R/Arr-3 interaction (I_max_ ≥ 100%) with a Hill slope close to −1, which indicates that the antagonist binds to the orthosteric site of the receptor (competitive antagonism) (Table 1). Conversely, the effects of gallamine on agonist-induced translocation of Arr-2 or Arr-3 exhibited two classical properties of NAM: a partial maximal inhibition (I_max_ < 100%) (“ceiling” effect) and a Hill slope less than −1, which is consistent with a negative cooperative effect (Figure 1C,D and Table 1).

As regards the orthosteric and allosteric inhibition of agonist-induced arrestin recruitment to the M_2_R, none of the pharmacodynamic parameters (I_max_, IC_50_, or Hill slope) were significantly different between HEKT/Arr-2 and HEKT/Arr-3 cells. This suggests that no significant functional differences appear to exist between the two isoforms of β-arrestin.

### 3.2. Effect of Serum M_2_ Muscarinic Receptor Autoantibodies on β-Arrestin Recruitment

**Figure 2:**
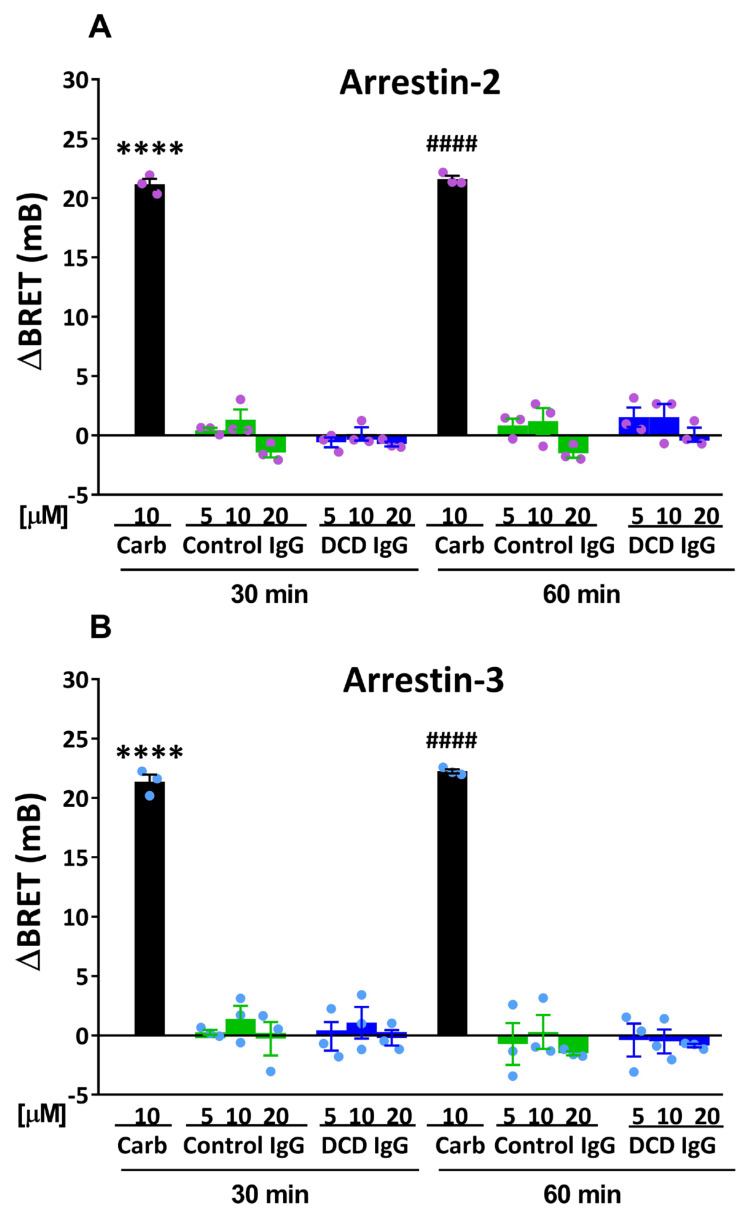
Interaction between M_2_R and β-arrestins in the presence of serum anti-M_2_R antibodies from patients with Chagas disease and dysautonomia (DCD patients). HEKT/Arr-2 (**A**) or HEKT/Arr-3 cells (**B**) were incubated with 10 µM carbachol or IgG from control individuals (Control IgG) or IgG from DCD patients (DCD IgG) at various concentrations for 30 or 60 min at 25 °C. BRET signals were determined as indicated in Figure 1. Net changes in BRET are shown as ΔBRET values and expressed in mB, where ΔBRET represents the BRET ratio in the presence of agonist or IgG fractions minus the BRET ratio in the presence of KRH buffer alone. Each bar represents the mean ± SEM of three Control or DCD IgG fractions or three experiments with carbachol performed at least in triplicate. Data were analyzed by two-way ANOVA followed by a post hoc Bonferroni test. Significant differences were observed compared to cells treated with Control or DCD IgG fractions for 30 min (**** *p* < 0.0001) (**A**) or 60 min (#### *p* < 0.0001) (**B**).

Before investigating whether anti-M_2_R AAb could modulate agonist-induced β-arrestin recruitment to the M_2_R, the ability of these AAb to enhance M_2_R/β-arrestin interaction in the absence of agonist was assessed. Therefore, the effects of IgG fractions from three different DCD patients or control volunteers on the translocation of Arr-2 versus Arr-3 were compared by performing BRET assays on HEKT/Arr-2 and HEKT/Arr-3 cells. As shown in Figure 2, carbachol (10 µM) induced Arr-2 or Arr-3 translocation to the M_2_R, as measured by an increase in the BRET signal between M_2_R-RLuc and Arr-2-YFP (or Arr-3-YFP), which ranged from 21 to 23 mB after 30 min or 60 min of agonist treatment. In contrast, Control IgG or DCD IgG, at varying concentrations, failed to enhance the BRET signal after treatment for either 30 or 60 min. These data indicate that M_2_R AAb from DCD patients do not stimulate the interaction between the M_2_R and Arr-2 or Arr-3 in the absence of an agonist.

### 3.3. Allosteric Modulation of Agonist-Induced β-Arrestin Recruitment by M_2_ Muscarinic Autoantibodies

**Figure 3:**
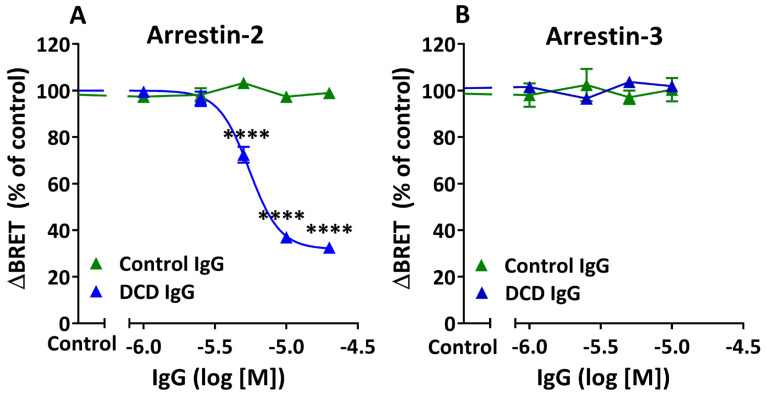
Differential effects of serum IgG anti-M_2_R antibodies from DCD patients on carbachol-induced β-arrestin recruitment. HEKT/Arr-2 (**A**) or HEKT/Arr-3 (**B**) cells were preincubated with Control or DCD IgG at varying concentrations for 30 min at 25 °C and then treated with carbachol (10 µM) for an additional 20 min before BRET recordings. Net changes in the BRET ratio were determined as ΔBRET and expressed in mB, as described in Figure 1. Data are shown as a percentage of control ΔBRET values, which correspond to cells treated with carbachol after preincubation with KRHA buffer alone. Data shown are the mean ± SEM of three Control or DCD IgG fractions tested in both HEKT/Arr-2 and HEKT/Arr-3 cell systems. Differences between Control and DCD IgG fractions were analyzed using two-factor ANOVA followed by a Bonferroni post hoc test. ((**A**) **** *p* < 0.0001; (**B**) NS). Data showing the effect of DCD IgG on carbachol-induced M_2_R/Arr-2 translocation was best fit to a sigmoidal function using nonlinear regression analysis.

The abilities of IgG fractions from three different control or DCD patients to modulate agonist-induced Arr-2 vs. Arr-3 translocation to the M_2_R were compared using the same BRET cell systems as before. HEKT/Arr-2 or HEKT/Arr-3 were first preincubated with Control IgG or DCD IgG at varying concentrations for 30 min and then treated with carbachol for 20 min before BRET determinations. Control IgG was unable to modulate the interaction of M_2_R with either Arr-2 or Arr-3 in the presence of carbachol at all concentrations tested (Figure 3A,B). In contrast, DCD IgG promoted a concentration-dependent decrease in agonist-induced Arr-2 translocation. However, the same DCD IgG fractions failed to inhibit agonist-induced Arr-3 recruitment (Figure 3A,B). Data from DCD IgG in Figure 3A were fit to a sigmoid curve using nonlinear regression analysis, which revealed pharmacodynamics parameters I_max_ = 68.1 ± 2.4%, pIC_50_ = 5.26 ± 0.02, and Hill slope = −4.03 ± 0.39. A submaximal inhibitory value and a Hill slope less than -1 are consistent with negative allosteric modulation. Therefore, these data suggest that anti-M_2_R AAb promote allosteric inhibition of agonist-induced Arr-2 recruitment to the target muscarinic receptor. Because agonist-induced M_2_R/Arr-3 interaction is unchanged by anti-M_2_R AAb, we conclude that the negative allosteric modulation of agonist-induced β-arrestin recruitment is biased towards Arr-2.

### 3.4. Epitope Specificity of the Negative Allosteric Modulation by M_2_ Muscarinic Autoantibodies

**Figure 4:**
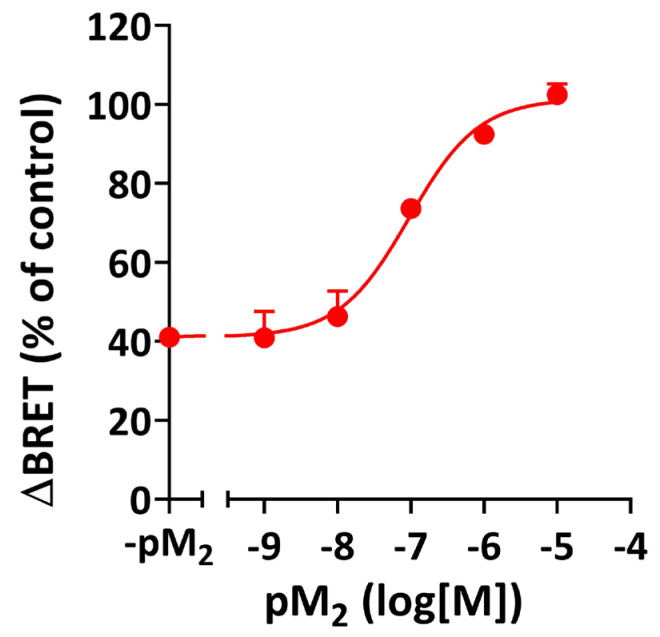
Inhibitory effect of IgG autoantibodies from DCD patients on Arr-2 recruitment to the M_2_R. Epitope specificity. Aliquots of serum IgG fractions from DCD patients (100 µM) were mixed with the muscarinic peptide pM_2_ at different concentrations (10 nM–100 µM) and incubated at 37 °C for 30 min. Then, HEKT/Arr-2 cells were preincubated at 25 °C for 30 min in the presence or absence of DCD IgG/pM_2_ cocktails diluted 1:10 in KRHA buffer, and further treated with carbachol (10 µM) for an additional 20 min at the same temperature. After BRET determinations, ΔBRET values were calculated and expressed in mB, as described in Figure 1. Data are shown as a percentage of the control ΔBRET value, which corresponds to cells treated with carbachol after preincubation with KRHA buffer alone (control ΔBRET value: 23.0 ± 0.6 mB, *n* = 3). Each data point represents the mean ± SEM of three IgG fractions from different DCD patients, performed at least in triplicate. Data were best fit to a sigmoidal function using nonlinear regression analysis.

The common allosteric binding site for muscarinic allosteric ligands at the M_2_R involves an acidic amino acid sequence (EDGE) within the second extracellular loop (II-ECL) [18]. To demonstrate that the negative allosteric activity of DCD IgG is mediated by the interaction of AAb with the allosteric binding site of the human M_2_R, we assessed the ability of a 25-mer peptide carrying the amino acid sequence of the II-ECL (pM_2_) to neutralize DCD IgG-mediated inhibition of agonist-induced Arr-2 recruitment using the above-mentioned BRET assay. Preincubation of DCD IgG with increasing amounts of pM_2_, followed by cell treatment with DCD IgG/pM_2_ complexes, resulted in a pM_2_ concentration-dependent attenuation of the inhibitory activity of DCD IgG on agonist-induced Arr-2 recruitment, which reached complete neutralization of AAb-mediated inhibitory activity at [pM_2_] = 10 µM (Figure 4). Nonlinear analysis of the data revealed pharmacodynamic parameters: Emax (101 ± 4%), pCE_50_ (7.04 ± 0.16), and Hill Slope (0.91 ± 0.25). These data demonstrate that the negative allosteric activity of DCD IgG is mediated by the interaction of anti-M_2_R AAb with the allosteric binding site of the M_2_R.

### 3.5. Allosteric Modulation by M_2_ Muscarinic Autoantibodies: Dependence on the Timing and Duration of Exposure of M_2_ Muscarinic Receptors to Specific Autoantibodies

**Figure 5:**
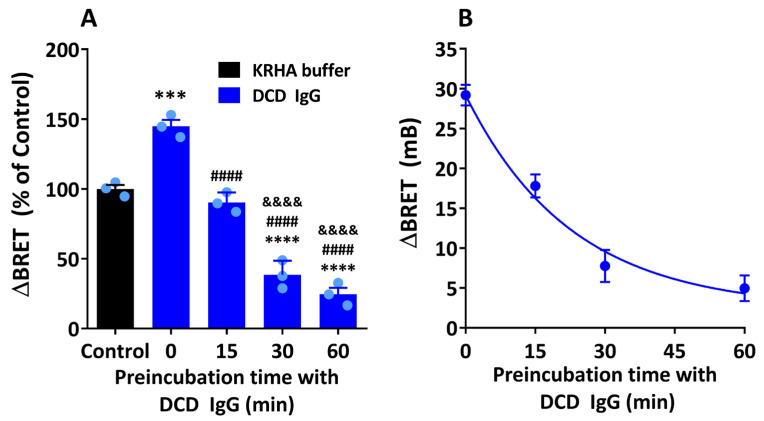
Effect of anti-M_2_R muscarinic antibodies on agonist-induced Arr-2 recruitment. Influence of preincubation time with DCD IgG. HEKT/Arr-2 cells were preincubated with buffer alone or DCD IgG (10 µM) for 0, 15, 30, or 60 min at 25 °C. Cells were then treated with carbachol (10 µM) for an additional 20 min before BRET recordings. Net changes in the BRET ratio were calculated as ΔBRET values and expressed in mB (**B**). Alternatively, data are presented as a percentage of the control ΔBRET value, which corresponds to cells treated with carbachol after preincubation with KRHA buffer alone (**A**). Each data point represents the mean ± SEM of three IgG fractions from different DCD patients or three assays with carbachol alone (control) performed in triplicate. Mean values were compared using one-way ANOVA followed by a Bonferroni multicomparison test. (**A**) Significant differences: **** vs. Control (*p* < 0.0001); *** vs. Control (*p* < 0.001); #### vs. 0 min (*p* < 0.0001); &&&& vs. 15 min (*p* < 0.0001). Time 0 min corresponds to cells preincubated with a mixture of DCD IgG and carbachol. (**B**) A plot of ∆BRET vs. preincubation time, ranging from 0 to 60 min, yielded an exponential decay curve, indicating a good fit to a first-order decay model (R^2^ = 0.97).

Previous reports have shown that the simultaneous exposure of M_2_R from an isolated heart preparation to both ACh and serum anti-M_2_R AAb resulted in an enhanced efficacy of ACh in promoting a negative chronotropic response [17]. These findings suggest that M_2_ muscarinic AAb behave as PAM of ACh-mediated responses. In contrast, the present study shows that the exposure of M_2_R to anti-M_2_R, AAb, followed by the addition of carbachol, results in an inhibition of agonist-induced Arr-2 recruitment, which suggests that these AAb can also act as NAM. Based on these results, we next examined whether the direction of allosteric modulation of agonist-induced receptor activation depends on the timing and duration of the exposure of M_2_R to AAb using our BRET-based assay. HEKT/Arr-2 cells were preincubated with DCD IgG for 0, 15, 30, or 60 min, and further treated with 10 µM carbachol for an additional 20 min. Cells treated with a mixture of 10 µM DCD IgG and 10 µM carbachol for 20 min (preincubation time with DCD IgG = 0 min) yielded a 44.9 ± 3.1% increase in BRET as compared with those treated with 10 µM carbachol alone. However, when the duration of preincubation was gradually increased up to 60 min, a time-dependent decrease in agonist-induced Arr-2 recruitment was observed (Figure 5A). Data were best fit to an exponential one-phase decay curve with a half-life value of 15.79 ± 0.01 min and a rate constant (K) value of 0.044 ± 0.008 min^−1^ (Figure 5B). The inhibitory effect of DCD IgG was 75.4 ± 4.6% at 60 min of preincubation time. This value was not significantly different from the inhibitory effect at 30 min (61.5 ± 5.8%). Therefore, the preincubation time with human DCD IgG used throughout this study was 30 min. These data indicate that anti-M_2_R AAb can enhance carbachol-induced Arr-2 recruitment when AAb and the muscarinic agonist act simultaneously. However, the exposure of M_2_R to M_2_ muscarinic AAb for at least 30 min before agonist addition results in a time-dependent inhibition of agonist-induced Arr-2 recruitment. In summary, the allosteric modulation of agonist-induced Arr-2 translocation depends on the timing and the duration of the exposure of M_2_R to specific AAb.

### 3.6. Inhibition of Agonist-Induced β-Arrestin Recruitment by M_2_ Muscarinic Autoantibodies: Allosteric Mechanism

**Figure 6:**
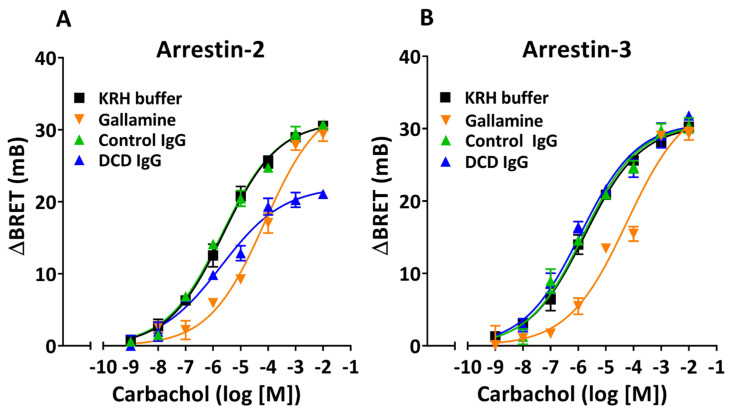
Inhibition of agonist-induced β-arrestin recruitment by anti-M_2_R muscarinic antibodies from DCD patients. Characterization of the allosteric mechanism. HEKT/Arr-2 (**A**) or HEKT/Arr-3 (**B**) were preincubated in the presence or absence of 10 µM Control or DCD IgG for 30 min at 25 °C. Alternatively, cells were preincubated in the presence of 100 µM gallamine for 15 min at the same temperature. Following either approach, cells were treated with carbachol at various concentrations for an additional 20 min. Net changes in the BRET ratio were calculated as ΔBRET values and expressed in mB, as indicated in Figure 1. Data shown in (**A**,**B**) are the mean ± SEM of three independent experiments (buffer alone or gallamine) or three IgG fractions, performed at least in triplicate. All curves were best fit to a sigmoidal function using nonlinear regression analysis, which provided the pharmacodynamic parameters shown in Table 2.

To get more insight into the mechanism underlying the negative allosteric modulation of agonist-induced M_2_R activation by anti-M_2_R AAb, we assessed the effect of DCD IgG on the agonist’s potency and efficacy in stimulating Arr-2 vs. Arr-3 recruitment. Thus, we performed BRET assays on HEKT/Arr-2 or HEKT/Arr-3 cells preincubated with buffer alone, DCD IgG, Control IgG, or gallamine, and further treated with carbachol at varying concentrations. Nonlinear regression analysis of agonist concentration–response (C-R) curves shows that preincubating cells with gallamine shifts the agonist curve to the right in both HEKT/Arr-2 and HEKT/Arr-3 cells, exhibiting higher EC_50_ values without significant changes in E_max_ values, as compared with control curves (KRHA buffer alone) (Figure 6A,B, Table 2). These decreases in agonist’s potency are consistent with the well-known pharmacodynamic behavior of gallamine as a NAM of agonist affinity. Control IgG does not promote significant changes in agonist-induced Arr-2 or Arr-3 recruitment. Thus, the Emax and EC_50_ values are similar to those from control curves in both BRET cell systems. In HEK-Arr-2 cells, DCD IgG promotes a significant decrease in E_max_ but not in EC_50_. Such a noncompetitive inhibitory effect is typical of a negative allosteric ligand that can inhibit the efficacy of an agonist without modifying its potency. In contrast, DCD IgG promotes no changes in agonist-induced Arr-3 translocation in either potency or efficacy, which confirms the previous observation that anti-M_2_R AAb cannot inhibit Arr-3 recruitment (Figure 3B).

## 4. Discussion

Evidence of a strong association between the presence of circulating anti-M_2_R antibodies and the presence of cardiovascular abnormalities and/or digestive disorders in *T. cruzi*-infected patients suggests that this antibody fraction could play a pathophysiological role in Chagas disease [29]. Over the years, several studies have demonstrated the intrinsic muscarinic activity of these autoantibodies in target organs associated with these disorders, such as the heart [5,9,12,13], esophagus [10], and colon [11] preparations, thereby supporting these hypotheses. Moreover, most studies on the pharmacological effects of muscarinic AAb to date have focused on their agonistic capacity, leading to the activation of Gi protein-mediated signal transduction pathways [9,10,11,30].

### 4.1. Intrinsic Activity of M_2_ Muscarinic Receptor Autoantibodies

In the present study, we examined whether these agonist-like anti-M_2_R AAb could enhance β-arrestin recruitment to the M_2_R. The fact that DCD IgG does not stimulate the interaction between the M_2_R and Arr-2 or Arr-3 may have various implications: (a) The interaction between the M_2_R with specific AAb generates an active state in the receptor molecule that favors G_i_ protein-activation but not β-arrestin translocation [5,15,24,30]. This suggests that M_2_ muscarinic AAb act as biased ligands with intrinsic muscarinic activity towards G_i_ protein signaling. (b) Given that agonist-induced M_2_R desensitization is mediated by β-arrestin recruitment to the phosphorylated receptor [31], the present findings are in agreement with previous data indicating that short-term exposure of M_2_R to serum anti-M_2_R AAb does not result in receptor desensitization [15,16,32]. (c) Because agonist-induced internalization of M_2_R can be arrestin-dependent [31,33], the present results are consistent with previous data demonstrating that anti-M_2_R AAb cannot promote M_2_R arrestin-dependent internalization [15,17].

### 4.2. Arr-2-Biased Modulation of β-Arrestin Recruitment by M_2_ Muscarinic Receptor Autoantibodies

The exposure of cellular M_2_R to anti-M_2_R AAb, followed by the addition of a muscarinic agonist, not only promotes a selective inhibition of Arr-2 recruitment but also the attenuation of G_i_ activation [15]. This suggests that the AAb/M_2_R interaction promotes a conformational change in the receptor molecule, which results in an impaired active state upon agonist binding. The ternary AAb/M_2_R/agonist complex does not promote G_i_ protein activation or Arr-2 recruitment as efficiently as the binary agonist/M_2_R complex does. In contrast, the ternary complex does not hinder Arr-3 translocation, which occurs as efficiently as it would in the absence of AAb. This suggests that the inhibitory effect of AAb on β-arrestin recruitment is biased towards Arr-2.

### 4.3. M_2_ Muscarinic Receptor Autoantibodies as Negative Allosteric Modulators

The most critical finding in the present study is that the inhibitory effect of anti-M_2_R AAb from CD patients on Arr-2 recruitment is allosteric in nature: (a) The inhibitory activity of muscarinic AAb is neutralized by a synthetic peptide carrying the amino acid sequence of the II-ECL (Figure 4). Because the II-ECL is part of the allosteric binding site of the M_2_R, these data indicate that anti-M_2_R AAb exert their inhibitory effect by interacting with the allosteric site of its target receptor. (b) The inhibition curve shows a partial inhibitory effect (I_max_ < 100%), which reveals saturation of allosteric sites, and a Hill Slope less than -1, which reflects a negative cooperative effect [34]. These values are consistent with negative allosteric modulation (Figure 3A). (c) According to classical pharmacodynamics, NAM can promote a decrease in agonist affinity for the orthosteric site and/or a decrease in agonist efficacy [35]. These changes result in rightward and/or downward shifts in agonist concentration–response curves, respectively. Our carbachol curves in the presence of DCD IgG indicate that the exposure of M_2_R to specific AAb, followed by agonist treatment, results in a decrease in agonist efficacy, but not in potency (Figure 6A and Table 2). As stated by Kenakin et al., when assay conditions provide a low receptor density (as in our BRET system), the chances of having a significant receptor reserve are negligible. In such a condition, a decrease in E_max_ without an increase in EC_50_ suggests a reduction in agonist efficacy, but not in affinity (noncompetitive effect) [35]. This conclusion is supported by the fact that anti-M_2_R AAb do not promote inhibition of Arr-3 recruitment to activated M_2_R, since an otherwise hypothetical reduction in agonist’s affinity generated by AAb should have also promoted a decrease in agonist potency, thereby shifting the agonist curve to the right (Figure 6B). Finally, the negative allosteric inhibition effect of anti-M_2_R AAb on agonist-induced receptor activation demonstrated in this study is also supported by previous results that had once proposed a partial agonistic activity for these AAb. In those studies, preincubation of rat atria with DCD IgG, its corresponding F(ab′)_2_ fraction, or monospecific anti-M_2_R IgG (anti-II-ECL) exerted non-competitive inhibition of agonist-induced negative inotropic effects in isolated heart assays [5,24,30].

### 4.4. Crosslinking-Mediated Negative Allosteric Modulation by M_2_R Autoantibodies

In a previous study, we reported that anti-M_2_R AAb from DCD patients could modestly enhance the BRET signal between M_2_R-Rluc and M_2_R-YFP in intact HEK 293T cells [20]. Given that the functional M_2_R in the cell membrane appears to be primarily a tetramer [36], the enhancing effect in BRET was interpreted as an antibody-induced conformational rearrangement within the M_2_R oligomer. The Fab fragment from DCD IgG was unable to mimic the effect of the bivalent AAb unless this bivalent structure was recreated in the presence of an anti-human Fab IgG antibody. These data suggest that anti-M_2_R AAb can crosslink adjacent receptors involved in preformed dimers or oligomers and stabilize pre-established M_2_ receptor/receptor interactions. Years later, we found that these AAb could inhibit agonist-induced Arr-2 recruitment, and that this effect is mediated by receptor crosslinking [15]. Based on these results, we conclude that a crosslinking-mediated conformational rearrangement within the antibody-bound receptor oligomer results in an altered active state upon agonist binding, leading to an impairment of M_2_R-associated signal transduction pathways. In other words, the negative allosteric modulation of agonist-induced M_2_R activation by anti-M_2_R AAb appears to be mediated by receptor crosslinking [37].

### 4.5. Time-Dependent Direction of Allosteric Modulation by M_2_ Muscarinic Autoantibodies

When evaluating the effect of the exposure time of HEKT/Arr-2 cells to anti-M_2_R autoantibodies on agonist-induced Arr-2 recruitment, two modulatory effects—opposite in direction and different in rate and magnitude—were observed: (a) a rapid 45% enhancement of BRET resulting from a 20 min cell treatment with a combination of DCD IgG and carbachol added simultaneously; (b) an exponential decline in BRET that reaches a maximum 75% inhibition after cell preincubation with DCD IgG for 60 min and an additional 20 min incubation after addition of carbachol. Regarding the first approach (a), our results are consistent with data published by Hernandez et al., who reported that anti-M_2_R AAb can exert positive allosteric modulation of acetylcholine-induced negative chronotropism after 4 min incubation in an isolated rabbit heart preparation [17]. Interestingly, these authors show that AAb can potentiate the ACh response at agonist concentrations exceeding the EC_50_, a finding similar to that observed in the present study. In contrast, the second approach (b) reveals a gradual inhibitory effect of AAb on agonist-induced Arr-2 recruitment, which requires a preincubation time of at least 30 min to become apparent. Considering all functional allosteric effects triggered by the IgG fraction from CD patients reported to date, we conclude that anti-M_2_R AAb can function as positive or negative allosteric modulators of agonist-induced M_2_R activation. Through this mechanism, M_2_ muscarinic AAb can either enhance or hinder the ability of the muscarinic agonist to promote G_i_ protein activation and Arr-2 recruitment [15,17], depending on the timing of AAb application with respect to carbachol addition (simultaneously or before) and the duration of M_2_R exposure to AAb.

### 4.6. Implications of the Present Findings in the Pathophysiology and Therapeutics of Chagas Disease

Anti-M_2_R AAb can bind to and activate cardiac M_2_R, leading to G_i_ protein activation, which can trigger negative inotropic and chronotropic effects [5,24,30]. However, in this report, we show that these AAb cannot stimulate Arr-2 or Arr-3 recruitment to the M_2_R, which could otherwise promote short-term receptor regulation (desensitization of the AAb-mediated agonist-like response and/or internalization of the AAb-bound M_2_R). This scenario generates an unregulated muscarinic response triggered by these AAb, which appears to play a pathogenic role in cardiac manifestations such as sinus node dysfunction [9] and genesis of the heterogeneity of ventricular repolarization and arrhythmia [12].

Negative allosteric modulation of M_2_R by M_2_ muscarinic AAb is particularly relevant in the context of dysautonomia secondary to CD. In the chronic phase of *T. cruzi* infection, cardiac M_2_R undergoes sustained exposure to anti-M_2_R AAb. In this scenario, a significant population of M_2_R will form M_2_R/AAb complexes. When ACh is released by cardiac parasympathetic nerve terminals, it will interact with AAb-bound M_2_R binary complexes. Upon ACh binding, the impaired active state in the ternary complexes would promote attenuated Gi protein activation (and Arr-2 recruitment), but unrestricted Arr-3 translocation. Therefore, a decreased efficacy of ACh in stimulating Gi protein signal transduction, combined with Arr-3-mediated M_2_R desensitization and internalization, would result in a reduction in ACh-induced negative chronotropic response, leading to parasympathetic dysautonomia.

Several strategies have been developed to counteract the detrimental effects of pathogenic AAb against GPCR. In particular, approaches aimed at disrupting the interaction between M_2_R and AAb, such as the use of synthetic peptides and aptamers, have proven effective in interfering with this interaction in both in vitro neutralization bioassays and in vivo preclinical mouse models [38,39,40]. The fact that conventional muscarinic allosteric ligands and functional AAb anti-M_2_R compete for binding to the same allosteric site within the target receptor suggests that the administration of muscarinic allosteric ligands could block the AAb/GPCR interaction, and thereby prevent AAb-mediated harmful effects [41]. For instance, Hernandez et al. have shown that the NAM gallamine can abolish the enhancement of ACh-induced negative chronotropic triggered by anti-M_2_R AAb on isolated rabbit hearts [17]. A few years later, Beltrame et al. demonstrated that gallamine can inhibit the enhancing effect of these AAb on M_2_ muscarinic receptor/receptor interaction [20]. The enhanced interaction between two protomers involved in the M_2_ muscarinic oligomer in the presence of anti-M_2_R AAb reflects the crosslinking-mediated conformational change in the receptor molecule, which results in the negative allosteric modulation of agonist-induced M_2_R activation [15]. In summary, these studies provide evidence suggesting that a muscarinic allosteric ligand, by interacting with the allosteric site of the M_2_R, can block the binding of anti-M_2_R AAb and thereby attenuate both positive and negative allosteric effects exerted by these AAb.

The use of computational methods like molecular dynamics simulations and ligand docking to compare the interactions between the human M_2_R and monoclonal antibodies against cross-reacting *T. cruzi* antigens with those involving this receptor and muscarinic allosteric ligands could reveal new aspects of the pharmacological nature of M_2_R/AAb interactions and the ability of muscarinic allosteric ligands to neutralize them [42,43,44].

Apart from Chagas disease, a large number of other medical conditions have been associated with the impaired autonomic control of cardiac or digestive function. Postural orthostatic tachycardia syndrome (POTS) is a particular type of dysautonomia characterized by an autonomic imbalance at the cardiovascular or digestive level (enhanced sympathetic activity and an attenuated vagal response) [45]. POTS can be classified as a primary (idiopathic) syndrome or secondary to a preexisting or coexisting infectious disease, like coronavirus disease 2019 (viral), Lyme disease (bacterial), and babesiosis (parasitic), among other medical conditions [46]. Interestingly, circulating functional antibodies interacting with autonomic neurotransmitter receptors, such as AAb to α_1_-adrenergic receptors (AAb anti-α_1_AR), AAb anti-β_1_AR, and AAb anti-M_2_R, have been identified in patients with POTS [47,48]. These AAb can moderately activate their target receptor and modulate agonist activity. However, while the AAb anti-β_1_AR potentiates agonist activity, the AAb anti-α_1_AR and AAb anti-M_2_R exert negative modulation. Therefore, the authors suggest that the former receptors could promote an increased sympathetic activity, while the latter may induce parasympathetic impairment [48]. Based on the present work, we encourage future studies involving AAb to GPCR from patients with various medical conditions to explore the potential allosteric nature of the modulatory activity exerted by these AAb. Binding studies as well as physiological and biochemical assays can be used to confirm that AAb exhibit true allosteric effects [17,37]. Moreover, other biochemical or immunocytochemical assays should rule out that the apparent allosteric activity of functional anti-GPCR AAb is merely the result of other regulatory mechanisms, such as receptor desensitization/internalization promoted by agonist-like AAb [15,17].

## 5. Conclusions

The present study demonstrates that serum anti-M_2_R autoantibodies from patients with chronic Chagas disease do not stimulate the interaction between the M_2_R and nonvisual arrestins (Arr-2 or Arr-3) in HEK 293T cells expressing M_2_R. However, preincubation of these cells for at least 30 min with AAb and further treatment with carbachol results in a selective inhibition of Arr-2 translocation without modifying Arr-3 recruitment. Inhibition curves of carbachol-induced Arr-2 translocation by AAb show a submaximal effect and a Hill Slope deviation from unity. In addition, carbachol concentration–response assays exhibit a noncompetitive inhibitory effect on agonist-induced Arr-2 recruitment, but Arr-3 translocation remains unchanged. These data suggest that anti-M_2_R AAb can function as NAM of agonist efficacy, and that their role as NAM of agonist-induced β-arrestin recruitment is biased towards arrestin-2. These AAb can also act as PAM when they act simultaneously with carbachol, which demonstrates that the direction of allosteric modulation depends on the timing of AAb application and the exposure time of M_2_R to specific AAb.

## Figures and Tables

**Table 1:** Inhibition of agonist-induced β-arrestin recruitment by orthosteric and allosteric ligands. Pharmacodynamic parameters.

	Arrestin-2			Arrestin-3		
Ligand	pIC_50_	I_max_ (%)	Hill Slope	pIC_50_	I_max_ (%)	Hill Slope
Atropine	6.83 ± 0.10 *	108.5 ± 5.1 **	−0.82 ± 0.12 ****	6.94 ± 0.09 *	109.2 ± 4.4 ***	−0.76 ± 0.09 ****
Gallamine	6.16 ± 0.29	73.2 ± 4.9	−0.31 ± 0.05	6.24 ± 0.26	70.9 ± 4.0	−0.27 ± 0.03

Maximal Inhibitory effect (I_max_), negative log of the half-maximal inhibitory concentration (pIC_50_), and Hill slope values, as determined by nonlinear regression analyses of data shown in Figure 1C,D, are listed. Comparison of pharmacodynamic parameters between curves within each panel or between them was performed using the extra sum of squares F test. Data are presented as the mean ± SEM. Significant differences vs. gallamine-preincubated cells are indicated (* *p* < 0.05, ** *p* < 0.01, *** *p* < 0.001, **** *p* < 0.0001). pIC_50_, I_max_, and Hill slope were not significantly different between HEKT/Arr-2 and HEKT/Arr-3 cells.

**Table 2:** Allosteric inhibition of agonist-induced β-arrestin recruitment mediated by anti-M_2_R autoantibodies from DCD patients. Pharmacodynamic parameters.

	Arrestin-2		Arrestin-3	
Treatment	pEC_50_	E_max_ (mB)	pEC_50_	E_max_ (mB)
KRHA Buffer	5.63 ± 0.10	31.1 ± 0.8	5.77 ± 0.10	30.5 ± 0.8
Gallamine	4.13 ± 0.26 ****	34.2 ± 3.0	4.24 ± 0.32 ****	34.1 ± 3.5
Control IgG	5.67 ± 0.10	31.2 ± 0.8	5.81 ± 0.15	30.8 ± 1.2
DCD IgG	5.71 ± 0.22	22.1 ± 1.2 &&, &&&&	5.92 ± 0.17	30.8 ± 1.3

Maximal effect (E_max_) and negative log of the half-maximal effective concentration (pEC_50_) values, as determined by nonlinear regression analyses of data shown in Figure 6A,B, are listed. Comparison of parameter values among curves within each panel (A or B) was made using one-way ANOVA followed by a Bonferroni multiple comparison test. Significant differences: **** vs. cells preincubated with buffer alone, Control IgG or DCD IgG (*p* < 0.0001); && vs. cells preincubated with either KRHA buffer or Control IgG (*p* < 0.01); &&&& vs. cells preincubated with gallamine (*p* < 0.0001).

## Data Availability

The datasets generated and/or analyzed during the current study are available from the corresponding author upon reasonable request. The minimal dataset is included in the Supplementary Materials.

## Supplementary Materials

The following supporting information can be downloaded at https://www.mdpi.com/article/10.3390/cells14231857/s1. Minimal Dataset, Equations, Curve Fitting and Parameter Definitions.

## Abbreviations

The following abbreviations are used in this manuscript:

II-ECL	second extracellular loop
α_1_AR	α_1_ adrenergic receptor
βAR, β_1_AR, or β_2_AR	β, β_1_, or β_2_ adrenergic receptor(s)
AAb	autoantibody(ies)
ACh	acetylcholine
Anti-α_1_AR AAb	AAb against α_1_AR
Anti-βAR AAb, anti-β_1_AR AAb, or anti-β_2_AR AAb	AAb against βAR, β_1_AR, or β_2_AR
Anti-M_2_R AAb/M_2_R AAb	AAb against M_2_R
Arr-2 or Arr-3	arrestin-2 or arrestin-3
Arr-2-YFP or Arr-3-YFP	Arr-2 or Arr-3 fused to enhanced yellow fluorescence protein
BRET	bioluminescence resonance energy transfer
BSA	bovine serum albumin
CD	Chagas disease
C-R	concentration–response
Control IgG	IgG(s) from (a) noninfected individual(s)
DCD IgG	IgG(s) from (a) patient(s) with DCD
DCD	dysautonomia secondary to CD
ECG	electrocardiogram
ERI	ELISA reactivity index
FBS	fetal bovine serum
GPCR	G protein-coupled receptor
GRK2	G protein-coupled receptor kinase 2
KHRA	Krebs-HEPES-Ringer buffer supplemented with BSA
M_2_R	M_2_ muscarinic receptor(s)
M_2_R-RLuc	M_2_R fused to *Renilla* luciferase
NAM	negative allosteric modulator(s)
PAM	positive allosteric modulator(s)
PBS	phosphate-buffered saline
POTS	postural orthostatic tachycardia syndrome
RFU	relative fluorescence units
RLU	relative luminescence units
SD	standard deviation
*T. cruzi*	*Trypanosoma cruzi*
